# Environmental and climate cardiology: some environmental issues highlighted at the annual meetings of the German Society for Cardiology (DGK) from 2007 to 2023

**DOI:** 10.1007/s00059-023-05223-0

**Published:** 2023-11-20

**Authors:** Omar Hahad

**Affiliations:** 1 Department of Cardiology, Cardiology I, University Medical Center of the Johannes Gutenberg University Mainz, Langenbeckstraße 1, 55131 Mainz, Germany; 2 German Center for Cardiovascular Research (DZHK), Partner Site Rhine-Main, Mainz, Germany

*Environmental cardiology* [1] and *climate cardiology* [2] are emerging terms emphasizing the importance of changes in the environment and climate on cardiovascular health and disease. The consequences of unhealthy environments are outlined in recent reports from the World Health Organization (WHO; [3]) and the Global Burden of Disease (GBD) study [4]. The WHO estimates that unhealthy environments accounted for up to 12.6 million global deaths in 2012, with a vast number of these deaths related to cardiovascular causes [3]. Likewise, the GBD study indicates that diseases caused by all forms of pollution account for 268 million disability-adjusted life years [5]. Specifically, climate change-related events such as extreme heat are associated with increased cardiovascular risks [6].

As acknowledged by the Lancet Commission on pollution and health, chemical pollution is considered the most important environmental cause of disease and premature death globally [7]. Chemical pollution-induced diseases were responsible for an estimated 9 million premature deaths (or 16% of all deaths worldwide) in 2015 [7]. A leading risk factor is ambient air pollution, which reduces global average life expectancy by 2.9 years and outcompetes even the reduction caused by tobacco smoking (2.2 years; [8]). In total, 9 million premature global deaths per year were attributed to air pollution in the form of particulate matter with a diameter of ≤ 2.5 μm (PM_2.5_; [9]); in fact, noise pollution is recognized as the second leading environmental health risk factor in the European region [10, 11]. Traffic noise is ubiquitous with > 20% of the European population being exposed to levels exceeding the EU guideline value of 55 dB [12]. A report from the European Environmental Agency suggests that traffic noise accounts for 12,000 premature deaths and 48,000 cases of ischemic heart disease per year in Europe [12]. Importantly, these exposures add to the harms of other coexisting cardiovascular risk factors [13].

The emerging appreciation of the cardiovascular effects of environmental and climate changes on cardiovascular health was discussed at the annual meetings of the German Society for Cardiology (DGK) from 2007 to 2023 (https://dgk.org/dgk-kongresse/). We searched this database using the following terms: “environment (Umwelt)”; “climate (Klima)”; “pollution (Verschmutzung)”; “air pollution (Luftervschmutzung)”; “noise (Lärm)”; or “heat (Hitze)”; focusing on the section “Kardiovaskuläre Risikofaktoren & Prävention—Umweltfaktoren/Umweltverschmutzung” (“Cardiovascular Risk Factors & Prevention—Environmental Factors/Environmental Pollution”). The findings of the search results are summarized in Table 1. The search identified 34 (average of two per year) contributions that were clearly linked to the topics during the 17 years of the meeting proceedings, indicating that there is room for improvement, although trends in recent years show promise. However, it is possible that contributions related to environmental/climate changes were missed because they were not directly evident based on the title of the presentation, and because the search terms might be insufficient. It is also possible that there may be inconsistencies between the printed program and the online program.

**Table 1:** Environment- and climate change-related findings presented at the annual meetings of the German Society for Cardiology (*DGK*) from 2007 to 2023

Year	Title of the annual meeting	Number of results	Contribution (first author)	
2007	Cardiovascular Imaging	0	–	
2008	Reperfusion, Remodeling and Regeneration	0	–	
2009	Not available	0	–	
2010	Not available	0	–	
2011	Heart Failure and Regeneration	0	–	
2012	Interventional Cardiovascular Therapy	1	(1)	Intranasale Instillation von Dieselrußpartikeln reduziert endotheliale Progenitorzellen und verstärkt die Atherogenese bei Mäusen (J. Pöss)
2013	The Cardiomyopathies	5	(1)	Is urban particulate air pollution or road traffic noise responsible for the association of traffic proximity with subclinical atherosclerosis—Results from the Heinz Nixdorf Recall Study (H. Kälsch)
			(2)	Nighttime aircraft noise exposure causes endothelial dysfunction in healthy adults (F. P. Schmidt)
			(3)	Höhere Inzidenz akuter ST-Hebungsinfarkte in Stadtteilen mit geringem Sozialstatus Daten aus dem Bremer Herzinfarktregister (S. Seide)
			(4)	Mikropartikel und Stickoxide in der Luft: Wie misst man Luftverschmutzung und was davon ist gefährlich? (C. Markard)
			(5)	Endotheliale Effekte ultrafeiner Rußpartikel: Führen Dieselabgase zum Infarkt? (T. Thum)
2014	Cardiology 2020: From Intervention to Prevention and Regeneration	0	–	
2015	Heart Failure	2	(1)	KORA: Umwelt, Entzündung und kardiovaskuläres Risiko (A. Peters)
			(2)	Fluglärm und Stress (T. Münzel)
2016	Cardiovascular Medicine—High-Tech Medicine	0	–	
2017	Rhythm for Life	2	(1)	Gene-environment interaction at the ADAMTS7 coronary artery disease locus with LDL-cholesterol (P. Schindler)
			(2)	Acute Exposure to Air Pollution Particulate Matter Aggravates Experimental Myocardial Infarction in Mice by Potentiating Cytokine Secretion from Lung Macrophages (D. Wolf)
2018	Cardiology 2018—From Basic Science to High-Performance Medicine	4	(1)	Auswirkungen von Feinstaub- und Lärmbelastung (T. Münzel)
			(2)	Bedeutung umweltbezogener Stressoren für die Inzidenz von Herz-Kreislauferkrankungen (T. Münzel)
			(3)	Crucial role for Nox2 and sleep deprivation in aircraft noise-induced vascular and cerebral oxidative stress (S. Kröller-Schön)
			(4)	Annoyance to different noise sources is associated with atrial fibrillation in the Gutenberg Health Study (O. Hahad)
2019	Cardiovascular Inflammation	3	(1)	Time response for development of vascular oxidative stress, endothelial dysfunction and high blood pressure by aircraft noise exposure (S. Kalinovic)
			(2)	Noise pollution exacerbates the development of arterial hypertension via additive oxidative stress and impairment of NO signaling (K. Frenis)
			(3)	Activation of AMPK with AICAR as a preventive strategy against aircraft noise induced oxidative stress, endothelial dysfunction and vascular inflammation (M. Kvandova)
2020	Not available	0	–	
2021	Overcome Boundaries and Discover New Worlds	4	(1)	Influence of age and environmental pollution on COVID-19 infection and immunity (D. Hilfiker-Kleiner)
			(2)	Exercise prevents negative effects of aircraft noise via α1AMPK activation (M. Kvandova)
			(3)	Vascular protection by ablation of lysozyme M‑positive cells in a model of aircraft noise exposure (S. Steven)
			(4)	Feinstaub, Lärm und Klimawandel: Was sind die Fakten und was raten wir unseren Patientinnen und Patienten? (T. Münzel)
2022	New Spaces for Cardiovascular Healthcare	7	(1)	Umweltfaktoren und kardiovaskuläres Risiko—Was bedeutet das für die Praxis? (T. Münzel)
			(2)	Air pollution particulate matter (PM) promotes obesity and adipose tissue inflammation by impairing thermogenesis in mice (S. T. Abogunloko)
			(3)	Long-term effects of aircraft noise exposure on vascular oxidative stress, endothelial function and blood pressure: no evidence for adaptation or resilience mechanisms (S. Steven)
			(4)	Assoziation von Luftschadstoffkonzentrationen und wetterabhängiger Variablen mit der Inzidenz des akuten Myokardinfarktes in Berlin. Eine Studie des Berlin-Brandenburger Herzinfarktregisters (B2HIR) (I. de Buhr-Stockburger)
			(5)	Endothelial α1AMPK activation abolishes aircraft noise-induced cardiovascular oxidative stress and endothelial dysfunction (M. Kvandova)
			(6)	The synergistic/additive effects of aircraft noise exposure on cardiovascular complications in diabetes mellitus (D. Mihalikova)
			(7)	Fluglärm im Schlaf—Mechanismen für kardiovaskuläre Erkrankungen (T. Münzel)
2023	Heart Failure Epidemic: Investigating Mechanisms, Healing Hearts	6	(1)	Aircraft noise exposure aggravates cardiovascular complications in diabetes mellitus (D. Mihaliková)
			(2)	Acute cardiovascular events due to environmental triggers in patients with pre-existing atherosclerotic cardiovascular diseases in Bavaria (K. Lechner)
			(3)	Adverse health effects of combined exposure to urban particulate matter and aircraft noise in mice—insights from the cerebropulmonary-cardiovascular axis (M. Kuntic)
			(4)	Luftverschmutzung und Kardiovaskuläres Risiko (T. Münzel)
			(5)	Association of air pollution and mortality in individuals with high cardiovascular risk (R. Maitra)
			(6)	Mitigation of aircraft noise-induced vascular dysfunction and oxidative stress by exercise, fasting and pharmacological activation—molecular proof of a protective key role of endothelial α1AMPK (P. Stamm)

Results containing the search terms without presenting or clearly indicating an environmental-/climate change-related topic were not included (e.g., as in “Die lokale, ‘microenvironmental’ VEGF-A-Konzentration—nicht die Gesamtdosis—bestimmt die Induktion von funktionellem und stabilem Gefäßwachstum in Ischämie”)

An important question is: What can be done to increase contributions related to environmental and climate cardiology? Some important steps include (a) increasing the awareness of researchers and clinicians on the impact of environmental/climate changes on cardiovascular health, (b) forming task forces and expert groups to increase funding for preclinical and clinical studies, (c) promoting educational programs (high school to university level, including medical curricula) that integrate these topics with other social determinants of health, and (d) improving epidemiological studies in developed and developing countries on the impact of environmental and climate changes on cardiovascular outcomes. Important is the existence of non-academic platforms that promote focused multidisciplinary studies on the association between environment and/or climate changes on health and disease, such as the Helmholtz Munich—German Research Center for Health and the Environment or the Leibniz Research Institute for Environmental Medicine located in Düsseldorf (both in Germany).

There are important unanswered questions on the cardiovascular effects of climate/environmental changes, including: What are the best methods to identify patients at risk? Which instruments and diagnostic tools should be used in primary prevention to determine individual exposures and risks? Is it beneficial to introduce environmental exposures into established cardiovascular risk scores? What specific therapeutic and preventive measures are effective on an individual level? Are the risks greater in patients with other comorbidities? A new health discipline that combines evidence from environmental and climate cardiology to lower the burden of cardiovascular disease could produce benefits at a patient and population level.
